# Analysis of sex differences in open-water ultra-distance swimming performances in the FINA World Cup races in 5 km, 10 km and 25 km from 2000 to 2012

**DOI:** 10.1186/2052-1847-6-7

**Published:** 2014-02-22

**Authors:** Matthias Alexander Zingg, Christoph Alexander Rüst, Thomas Rosemann, Romuald Lepers, Beat Knechtle

**Affiliations:** 1 Institute of General Practice and for Health Services Research, University of Zurich, Zurich, Switzerland; 2 INSERM U1093, Faculty of Sport Sciences, University of Burgundy, Dijon, France; 3 Gesundheitszentrum St. Gallen, Vadianstrasse 26, 9001 St. Gallen, Switzerland

**Keywords:** Woman, Man, Athlete, Endurance, Performance, Water sport

## Abstract

**Background:**

The present study investigated the changes in swimming speeds and sex differences for elite male and female swimmers competing in 5 km, 10 km and 25 km open-water FINA World Cup races held between 2000 and 2012.

**Methods:**

The changes in swimming speeds and sex differences across years were analysed using linear, non-linear, and multi-level regression analyses for the annual fastest and the annual ten fastest competitors.

**Results:**

For the annual fastest, swimming speed remained stable for men and women in 5 km (5.50 ± 0.21 and 5.08 ± 0.19 km/h, respectively), in 10 km (5.38 ± 0.21 and 5.05 ± 0.26 km/h, respectively) and in 25 km (5.03 ± 0.32 and 4.58 ± 0.27 km/h, respectively). In the annual ten fastest, swimming speed remained constant in 5 km in women (5.02 ± 0.19 km/h) but decreased significantly and linearly in men from 5.42 ± 0.03 km/h to 5.39 ± 0.02 km/h. In 10 km, swimming speed increased significantly and linearly in women from 4.75 ± 0.01 km/h to 5.74 ± 0.01 km/h but remained stable in men at 5.36 ± 0.21 km/h. In 25 km, swimming speed decreased significantly and linearly in women from 4.60 ± 0.06 km/h to 4.44 ± 0.08 km/h but remained unchanged at 4.93 ± 0.34 km/h in men. For the annual fastest, the sex difference in swimming speed remained unchanged in 5 km (7.6 ± 3.0%), 10 km (6.1 ± 2.5%) and 25 km (9.0 ± 3.7%). For the annual ten fastest, the sex difference remained stable in 5 km at 7.6 ± 0.6%, decreased significantly and linearly in 10 km from 7.7 ± 0.7% to 1.2 ± 0.3% and increased significantly and linearly from 4.7 ± 1.4% to 9.6 ± 1.5% in 25 km.

**Conclusions:**

To summarize, elite female open-water ultra-distance swimmers improved in 10 km but impaired in 25 km leading to a linear decrease in sex difference in 10 km and a linear increase in sex difference in 25 km. The linear changes in sex differences suggest that women will improve in the near future in 10 km, but not in 25 km.

## Background

Performance trends in ultra-endurance sports disciplines have been investigated in running [1], cycling [2], triathlon [3,4] and more recently also in long-distance swimming [5-9]. Besides investigating general trends of performance, a widely discussed topic was the specific sex difference in ultra-endurance performance [1,10,11]. Open-water long-distance swimming is a relatively young sports discipline [12] compared to running where marathons exist for more than a century [13]. The first open-water long-distance swimmers were single athletes. On August 24, 1875, Captain Matthew Webb of Great Britain became the first man to successfully swim the ‘English Channel’ without assistance [14]. Not until 1986, open-water long-distance swimming was established as a World Cup discipline [15]. Later, open-water long-distance swimming was introduced in the 2008 Olympic Games in Beijing with a 10 km open-water competition which was again held in the 2012 Olympic Games in London [16]. Generally, long-distance swimming is defined as freestyle swimming for distances of longer than 400 m. The International Federation of Natation (FINA) furthermore differentiates between long-distance swimming in competitions of more than 400 m in freestyle and more than 200 m in other swimming disciplines and open-water swimming [15]. On a professional level, FINA lists 5 km, 10 km and 25 km as regular race distances [15]. However, any freestyle open-water swimming competition of more than 400 m would meet the required criteria [15].

The sex difference in open-water long-distance swimming has been investigated in a few studies [5-7,9], whereas more studies concentrated on classical pool swimming distances of 50 m to 1,500 m [17-21]. For example, in the 2000 Olympic Games, women were on average ~11% slower than men in different types of strokes and distances ranging from 50 m to 1,500 m [18]. Thibault et al. [19] reported a mean sex difference in performance of ~8.9% in swimming races in the 2008 Olympic Games in Beijing. Tanaka and Seals [20] reported a decrease in the sex difference in freestyle swimming performance with increasing race distance ranging from 50 m to 1,500 m whereas Eichenberger et al. [5-7] found larger sex differences in freestyle swimming performance in race distances longer than the 1,500 m freestyle. To date, the largest values for sex differences in swimming were reported for the split times in swimming in a Deca Iron ultra-triathlon where women were ~45% slower in 38 km freestyle swimming compared to men [22]. However, it might be argued that results in split times never reflect the reality of a single discipline, since swimming split times in a triathlon might be influenced by either cycling or running as subsequent disciplines.

Concerning the sex difference in open-water ultra-distance swimming, recent studies investigated the performances in the 26.4 km ‘Lake Zürich Swim’ [7], the 34 km ‘English Channel Swim’ [5,8] and the ’12 h Swim of Zürich’ [6]. In both the ‘Lake Zürich Swim’ [7] and the ‘English Channel Swim’ [8] men were faster than women. Depending from which side of the ‘English Channel’ the swim was accomplished the sex difference in swimming time differed from 6.7% for England-to-France to 8.9% for France-to-England [5]. Eichenberger et al. [5,7] stated that it would be unlikely that women would outperform men in ultra-distance swimming in the future. In the ’12 h Swim of Zürich’, however, women were able to accomplish similar distances as their male counterparts [6]. The comparison of different events is even more complicated as for example ‘the English Channel Swim’ is not a race but has to be completed by each athlete individually with a supporting crew [14]. Furthermore, in the ’12 h Swim of Zürich’ [6] and the ‘Lake Zürich Swim’ [7], mainly recreational athletes compete.

Single human accomplishments recently called the attention of the long-distance swimming community. In the summer of 2013, Diana Nyad [23] crossed the sea between Havana, Cuba, and Key West, Florida, as the first person ever without a shark cage. She accomplished the 177 km swim within 53 hours [23]. Another milestone in long-distance swimming history was set in 2013 when Christoph Wandratsch crossed the length of Lake Constance (66.7 km) in 20 h and 41 min [24]. As Diana Nyad covered a considerably longer distance at the nearly the same swimming speed (3.3 km/h) like Christoph Wandratsch (3.2 km/h), it could be argued that the world’s best long-distance swimmers would be women rather than men. However, the achievements of Christoph Wandratsch and Diana Nyad are not directly comparable due to different water temperatures. Therefore, real open-water ultra-distance swim competitions such as the ‘Lake Zürich Swim’ [25] are more suitable to investigate the sex difference in ultra-swimming performance than single achievements. However, the above mentioned competitions [5-7] investigated recreational swimmers and never included the entire world elite and more men than women competed in these events. The reported sex differences in ultra-swimming performance may therefore be biased by the number of finishers and the participation of non-professional athletes. Official events held in 5 km, 10 km and 25 km in the World Cup [15] would therefore be a better option to bypass these factors.

These recent controversial findings allow interpretations of both an increase in the sex difference in performance with increasing race distance or *vice versa*. However, to date, mainly recreational swimmers have been investigated in in the 26.4 km ‘Lake Zürich Swim’ [7], the 34 km ‘English Channel Swim’ [5,8] and the ’12 h Swim of Zürich’ [6] and there is a lack of data for elite swimmers. Additionally, the single performances of Diana Nyad and Christoph Wandratsch do not allow a generalization for the sex differences in ultra-distance swimming performance.

Since professionalism of athletes might influence the sex difference in performance [9], we intended to investigate the sex differences in swimming performance in 5 km, 10 km and 25 km open-water ultra-distance swimming races in professional athletes. Additionally, in a high-level swimming race, the fastest women may have the possibility to draft behind the fastest men. This might enable the fastest women to reduce the sex difference. The aims of the present study were therefore to investigate the changes in sex differences across years with increasing race distances from 5 km to 25 km. Based upon existing findings for recreational swimmers, we hypothesized for elite swimmers (*i*) that men would be faster than women from 5 km to 25 km, and (*ii*) the sex differences in performance would decrease with increasing race distance.

## Methods

### Ethics

All procedures used in the study met the ethical standards of the Swiss Academy of Medical Sciences [26] and were approved by the Institutional Review Board of Kanton St. Gallen, Switzerland, with a waiver of the requirement for informed consent of the participants given the fact that the study involved the analysis of publicly available data.

### Data sampling and data analysis

The data set for this study was obtained from the website of the FINA [15]. All athletes who ever finished a 5 km, 10 km and 25 km FINA World Cup open-water swim race between 2000 and 2012 were included. To determine the changes over time in peak swimming speed and in the sex difference in swimming speed, race times of the annual top and the annual top ten women and men were analysed. To increase the comparability between different race distances, all race times were converted to swimming speed (km/h) using the equation [swimming speed (km/h)] = [race distance (km)]/[race time (h)]. When less than the needed amount of athletes was available in a certain year for a certain distance, that year was excluded from analysis. To estimate the power density of the swimmers, the time differences between the last and the first finisher as well as between tenth and the first finisher were analysed and expressed as a percentage of the winner performance for both women and men.

### Statistical analysis

In order to increase the reliability of the data analyses, each set of data was tested for normal distribution and for homogeneity of variances prior to statistical analyses. Normal distribution was tested using a D’Agostino and Pearson omnibus normality test and homogeneity of variances was tested using a Levene’s test. Trends in participation were analysed using regression analysis with ’straight line‘ and ’exponential growth equation‘ model where for each set of data (*e.g.* each sex) both models where compared using Akaike’s Information Criteria (AICc) to decide which model showed the highest probability of correctness. Single and multi-level regression analyses were used to investigate changes across years in swimming speed and age of the athletes. A hierarchical regression model was used to avoid the impact of a cluster-effect on results in case one athlete finished more than once in the annual top or the annual top ten. Furthermore, regression analyses of swimming speed were corrected for age of the athletes to prevent a misinterpretation of an ‘age-effect’ as a ‘time-effect’. Since it is assumed that the change in sex difference in endurance performance is non-linear [27], we additionally calculated the non-linear regression model fitting the data best. We compared the best-fit non-linear models to the linear models using AIC and F-test in order to show which model would be the most appropriate to explain the trend of the data. Statistical analyses were performed using IBM SPSS Statistics (Version 21, IBM SPSS, Chicago, IL, USA), CurveExpert Professional (Version 2.0.3, Hyams D.G.) and GraphPad Prism (Version 6.01, GraphPad Software, La Jolla, CA, USA). Significance was accepted at *p* < 0.05 (two-sided for *t*-tests). Data in the text and figures are given as mean ± standard deviation (SD).

## Results

### Participation trends

**Figure 1:**
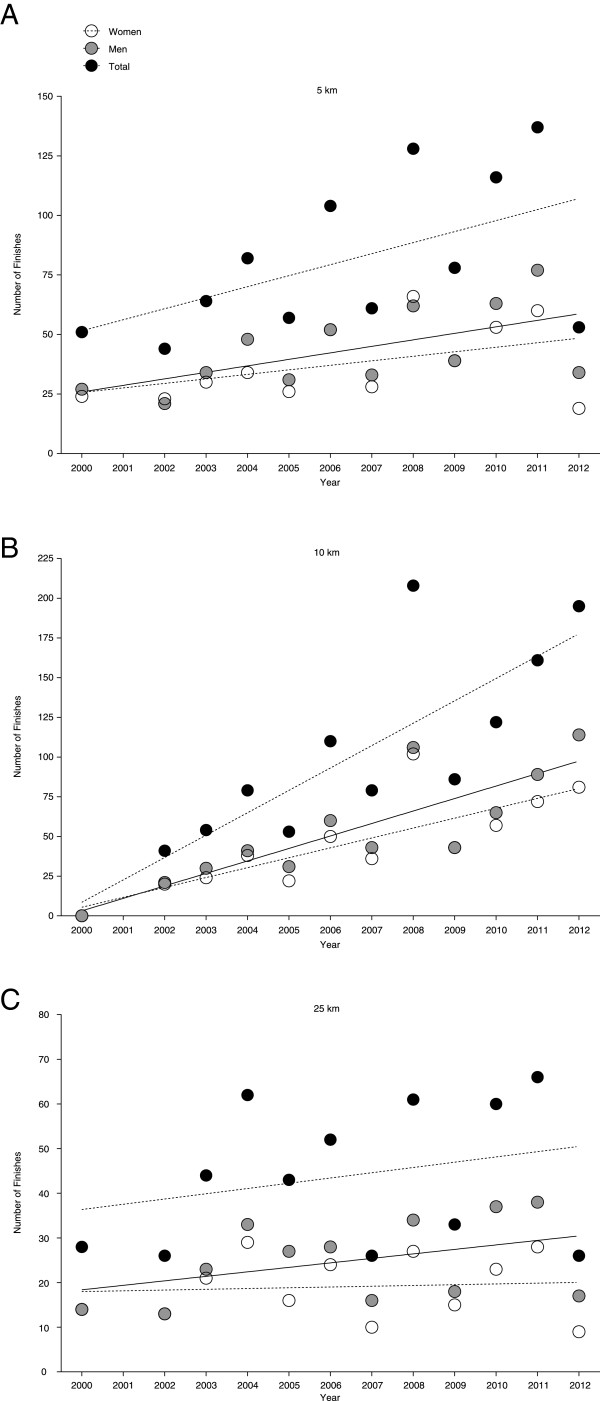
Number of finishes for women and men in 5 km (Panel A), 10 km (Panel B) and 25 km (Panel C).

Table 1 presents the numbers of both finishes and finishers. Most swimmers competed in 5 km, followed by 10 km and 25 km. Between 2000 and 2012, on average 38 ± 16 women and 43 ± 17 men competed in a 5 km, 45 ± 29 women and 57 ± 35 men in a 10 km, and 19 ± 7 women and 25 ± 9 men in a 25 km FINA World Cup event. The number of finishers in 5 km increased significantly for men (r^2^ = 0.36, *p* < 0.05) but not for women and overall finishers (*p* > 0.05) (Figure 1A). In 10 km, the number of finishers increased significantly for both women (r^2^ = 65, *p* = 0.0014) and men (r^2^ = 0.72, *p* = 0.0004) (Figure 1B). In 25 km, the overall number of finisher was unchanged (*p* > 0.05) (Figure 1C). Table 2 presents the numbers of finishes regarding the origin of the competitors. When all three race distances were considered, most of the finishes were achieved by competitors originating from Russia, followed by swimmers originating from Italy and Germany.

**Table 1:** Number of finishes and finishers in open-water swim races from 2000 to 2012

	**5 km**			**10 km**			**25 km**		
	**Women**	**Men**	**Overall**	**Women**	**Men**	**Overall**	**Women**	**Men**	**Overall**
**1 Finish**	118	155	273	89	127	215	53	78	130
**2 Finishes**	54	54	108	36	47	83	10	21	31
**3 Finishes**	17	22	39	24	27	51	6	8	14
**4 Finishes**	12	10	22	15	16	31	6	8	13
**5 Finishes**	5	5	10	13	10	23	5	5	11
**6 Finishes**	3	6	9	7	5	11	3	6	9
**7 Finishes**	2	3	5	6	6	13	3	1	4
**8 Finishes**	2	3	5	3	7	10	1		1
**9 Finishes**	2	1	3	1	1	2	2	1	3
**10 Finishes**		1	1	3	1	4		1	1
**> 10 Finishes**	3	2	5	3	6	9	2	3	5
**Finishes**	454	521	975	545	643	1,188	229	298	527
**Finishers**	218	262	480	200	253	452	91	132	222

**Table 2:** Number of finishes regarding the origin of the athletes

	**5 km**			**10 km**			**20 km**			
**Country**	**Women**	**Men**	**Overall**	**Women**	**Men**	**Overall**	**Women**	**Men**	**Overall**	**All**
RUS	37	41	78	37	42	79	37	33	70	454
ITA	41	41	82	38	41	79	31	33	64	450
GER	30	41	71	36	40	76	30	18	48	390
FRA	23	36	59	25	39	64	12	36	48	342
ESP	30	31	61	35	28	63	23	12	35	318
CZE	22	31	53	23	28	51	12	30	42	292
USA	18	17	35	21	20	41	14	13	27	206
AUS	16	18	34	20	19	39	12	15	27	200
GBR	21	23	44	24	23	47	0	4	4	190
HUN	18	20	38	21	24	45	5	4	9	184
UKR	12	20	32	21	25	46	2	6	8	172
CAN	15	15	30	16	19	35	6	8	14	158
BRA	17	17	34	17	20	37	2	2	4	150
NED	9	8	17	22	15	37	13	8	21	150
ECU	12	13	25	14	14	28	0	1	1	108
MEX	8	8	16	16	14	30	1	4	5	102
BUL	2	5	7	4	21	25	5	12	17	98
VEN	10	11	21	13	12	25	1	1	2	96
CRO	10	6	16	12	13	25	3	4	7	96
GRE	5	12	17	10	17	27	0	2	2	92
SLO	15	5	20	12	4	16	3	7	10	92
ARG	5	5	10	8	13	21	3	8	11	84
SUI	15	6	21	12	5	17	1	2	3	82
RSA	7	10	17	9	13	22	0	0	0	78
CHN	10	4	14	12	9	21	2	2	4	78
POR	1	8	9	6	16	22	1	5	6	74
EGY	4	4	8	4	15	19	0	7	7	68
ISR	4	6	10	2	17	19	0	4	4	66
BEL	3	1	4	3	11	14	5	2	7	50
HKG	3	2	5	8	5	13	0	0	0	36
MKD	0	3	3	0	4	4	2	7	9	32
NZL	3	2	5	7	4	11	0	0	0	32
POL	4	0	4	6	2	8	1	2	3	30
AZE	2	1	3	6	4	10	0	1	1	28
SVK	5	7	12	1	0	1	0	0	0	26
GUA	4	2	6	3	2	5	0	0	0	22
JPN	1	1	2	4	5	9	0	0	0	22
CRC	0	5	5	0	4	4	0	0	0	18
IRL	0	4	4	0	5	5	0	0	0	18
SWE	3	0	3	5	0	5	0	0	0	16
TUR	2	5	7	1	0	1	0	0	0	16
SYR	0	0	0	0	4	4	0	3	3	14
KAZ	0	1	1	0	4	4	0	1	1	12
INA	1	2	3	1	2	3	0	0	0	12
AUT	0	3	3	0	3	3	0	0	0	12
CHI	1	2	3	1	2	3	0	0	0	12
BLR	0	0	0	1	2	3	0	1	1	8
DOM	0	3	3	0	1	1	0	0	0	8
MNE	3	0	3	1	0	1	0	0	0	8
OMA	0	2	2	0	2	2	0	0	0	8
CYP	0	0	0	2	1	3	0	0	0	6
PUR	1	1	2	1	0	1	0	0	0	6
FAR	0	1	1	0	1	1	0	0	0	4
FIN	0	2	2	0	0	0	0	0	0	4
GUM	0	0	0	0	2	2	0	0	0	4
IND	0	0	0	0	2	2	0	0	0	4
MAS	0	0	0	2	0	2	0	0	0	4
PLE	0	1	1	0	1	1	0	0	0	4
SRB	1	1	2	0	0	0	0	0	0	4
TUN	0	0	0	0	2	2	0	0	0	4
UAE	0	2	2	0	0	0	0	0	0	4
BAN	0	1	1	0	0	0	0	0	0	2
COK	0	1	1	0	0	0	0	0	0	2
CUB	0	0	0	1	0	1	0	0	0	2
DEN	0	0	0	0	0	0	1	0	1	2
KSA	0	1	1	0	0	0	0	0	0	2
LBA	0	1	1	0	0	0	0	0	0	2
MAR	0	0	0	1	0	1	0	0	0	2
MCD	0	0	0	0	1	1	0	0	0	2
THA	0	1	1	0	0	0	0	0	0	2

RUS = Russia, ITA = Italy, GER = Germany, FRA = France, ESP = Spain, CZE = Czech Republic, USA = United States of America, AUS = Australia, GBR = Great Britain, HUN = Hungary, UKR = Ukraine, CAN = Canada, BRA = Brazil, NED = Netherlands, ECU = Ecuador, MEX = Mexico, BUL = Bulgaria, VEN = Venezuela, CRO = Croatia, GRE = Greece, SLO = Slovenia, ARG = Argentina, SUI = Switzerland, RSA = Republic of South Africa, CHN = China, POR = Portugal, EGY = Egypt, ISR = Israel, BEL = Belgium, HKG = Hong Kong, MKD = Macedonia, NZL = New Zealand, POL = Poland, AZE = Azerbaijan, SVK = Slovakia, GUA = Guatemala, JPN = Japan, CRC = Costa Rica, IRL = Ireland, SWE = Sweden, TUR = Turkey, SYR = Syria, KAZ = Kazakhstan, INA = Indonesia, AUT = Austria, CHI = Chile, BLR = Belarus, DOM = Dominican Republic, MNE = Montenegro, OMA = Oman, CYP = Cyprus, PUR = Puerto Rico, FAR = Faroe Islands, FIN = Finland, GUM = Guam, IND = India, MAS = Malaysia, PLE = Palestine, SRB = Serbia, TUN = Tunisia, UAE = United Arab Emirates, BAN = Bangladesh, COK = Cook Islands, CUB = Cuba, DEN = Denmark, KSA = Saudi Arabia, LBA = Libya, MAR = Morocco, ROU = Romania, THA = Thailand.

### Change in swimming speed across the years

**Figure 2:**
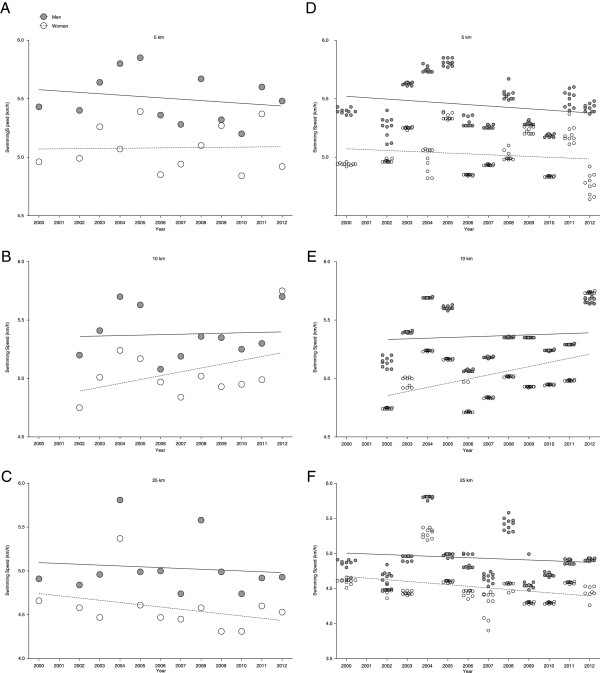
Changes in swimming speeds across years for the annual fastest women and men in 5 km (Panel A), 10 km (Panel B) and 25 km (Panel C) and for the annual ten fastest women and men in 5 km (Panel D), 10 km (Panel E) and 25 km (Panel F).

For the annual fastest, swimming speed remained stable across years in 5 km (5.50 ± 0.21 km/h for men and 5.08 ± 0.19 km/h for women) (Figure 2A), in 10 km (5.38 ± 0.21 km/h for men and 5.05 ± 0.26 km/h for women) (Figure 2B) and in 25 km (5.03 ± 0.32 km/h for men and 4.58 ± 0.27 km/h for women) (Figure 2C) also when corrected for multiple finishes and age of the athletes with multiple finishes (Table 3). Considering the annual ten fastest, swimming speed remained constant in 5 km in women (5.02 ± 0.19 km/h) but decreased significantly and linearly (Table 4) in men from 5.42 ± 0.03 km/h to 5.39 ± 0.02 km/h (Figure 2D) also when controlled for multiple finishes and age of the athletes with multiple finishes (Table 3). In 10 km, swimming speed increased significantly and linearly (Table 4) in women from 4.75 ± 0.01 km/h (2002) to 5.74 ± 0.01 km/h (2012) but remained stable in men at 5.36 ± 0.21 km/h (Figure 2E) also when controlled for multiple finishes and age of the athletes with multiple finishes (Table 3). In 25 km, swimming speed decreased significantly and linearly (Table 4) in women from 4.60 ± 0.06 km/h to 4.44 ± 0.08 km/h but remained unchanged at 4.93 ± 0.34 km/h in men (Figure 2F) also when controlled for multiple finishes and age of the athletes with multiple finishes (Table 3).

**Table 3:** Multi-level regression analyses for swimming speed of the annual fastest and the annual ten fastest swimmers (Model 1) with correction for multiple finishes (Model 2) and age of athletes with multiple finishes (Model 3)

**Model**	***ß***	**SE ( *ß *)**	**Stand. *ß***	**T**	***p***
		**Annual fastest swimmers**			
**5 km women**					
**1**	0.002	0.017	0.031	0.097	0.924
**2**	0.002	0.017	0.031	0.097	0.924
**3**	0.002	0.024	0.037	0.081	0.937
**5 km men**					
**1**	-0.012	0.017	-0.210	-0.678	0.513
**2**	-0.012	0.017	-0.210	-0.678	0.513
**3**	-0.008	0.019	-0.142	-0.413	0.689
**10 km women**					
**1**	0.033	0.024	0.409	1.346	0.211
**2**	0.033	0.024	0.409	1.346	0.211
**3**	0.048	0.038	0.591	1.250	0.247
**10 km men**					
**1**	0.004	0.021	0.061	0.183	0.859
**2**	0.004	0.021	0.061	0.183	0.859
**3**	0.082	0.031	1.278	2.622	0.031
**25 km women**					
**1**	-0.026	0.022	-0.352	-1.190	0.262
**2**	-0.026	0.022	-0.352	-1.190	0.262
**3**	-0.041	0.021	-0.560	-1.926	0.086
**25 km men**					
**1**	-0.009	0.027	-0.110	-0.349	0.734
**2**	-0.009	0.027	-0.110	-0.349	0.734
**3**	0.014	0.032	0.159	0.433	0.676
**Annual ten fastest swimmers**					
**5 km women**					
**1**	-0.007	0.005	-0.142	-1.559	0.122
**2**	-0.007	0.005	-0.142	-1.559	0.122
**3**	-0.006	0.005	-0.113	-1.236	0.219
**5 km men**					
**1**	-0.012	0.005	-0.214	-2.377	0.019
**2**	-0.012	0.005	-0.214	-2.377	0.019
**3**	-0.012	0.005	-0.218	-2.424	0.017
**10 km women**					
**1**	0.036	0.007	0.421	4.820	< 0.001
**2**	0.036	0.007	0.421	4.820	< 0.001
**3**	0.035	0.007	0.419	4.796	< 0.001
**10 km men**					
**1**	0.006	0.006	0.089	0.929	0.355
**2**	0.006	0.006	0.089	0.929	0.355
**3**	0.005	0.006	0.073	0.727	0.469
**25 km women**					
**1**	-0.023	0.007	-0.312	-3.548	0.001
**2**	-0.023	0.007	-0.312	-3.548	0.001
**3**	-0.023	0.007	-0.310	-3.498	0.001
**25 km men**					
**1**	-0.011	0.009	-0.114	-1.250	0.214
**2**	-0.011	0.009	-0.114	-1.250	0.214
**3**	-0.011	0.009	-0.120	-1.300	0.196

**Table 4:** Comparison of linear and non-linear regression analysis of changes in swimming speed across years to determine which model is the best

**Swimming speed**	**Kind of regression**	**Sum of squares**	**DOF**	**AICc**	**Best regression AIC-Test**	**Best regression F-Test**	**Delta**	**Probability**	**Likelihood**
Annual fastest men 5 km	Polynomial	0.26	5	-16.81	Linear	Linear	20.03	4.4 e^-05^	99.9%
	Linear	0.45	10	-36.85					
Annual fastest women 5 km	Polynomial	0.33	0	-21.12	Linear	Undetermined	16.50	0.00026	99.9%
	Linear	0.42	10	-37.63					
Annual fastest men 10 km	Polynomial	0.21	5	-21.46	Linear	Linear	11.26	0.0035	99.6%
	Linear	0.44	9	-32.72					
Annual fastest women 10 km	Polynomial	0.064	0	-36.51	Polynomial	Undetermined	6.81	0.032	96.7%
	Linear	0.59	9	-29.70					
Annual fastest men 25 km	Polynomial	0.98	0	-7.99	Linear	Undetermined	17.78	0.00013	99.9%
	Linear	1.14	10	-25.77					
Annual fastest women 25 km	Polynomial	0.49	0	-16.20	Linear	Undetermined	15.16	0.00050	99.9%
	Linear	0.71	10	-31.37					
Annual 10 fastest men 5 km	Polynomial	0.31	0	-21.52	Linear	Undetermined	11.17	0.0037	99.6%
	Linear	0.64	10	-32.69					
Annual 10 fastest women 5 km	Polynomial	0.26	5	-16.95	Linear	Linear	22.44	1.33 e^-05^	99.9%
	Linear	0.36	10	-39.39					
Annual 10 fastest men 10 km	Polynomial	0.15	0	-26.60	Linear	Undetermined	6.11	0.044	95.5%
	Linear	0.44	9	-32.71					
Annual 10 fastest women 10 km	Polynomial	0.12	4	-16.10	Linear	Linear	12.82	0.0016	99.8%
	Linear	0.63	9	-28.93					
Annual 10 fastest men 25 km	Polynomial	1.80	0	-0.72	Linear	Undetermined	16.52	0.00025	99.9%
	Linear	2.33	10	-17.24					
Annual 10 fastest women 25 km	Polynomial	0.78	0	-10.66	Linear	Linear	20.74	3.12 e^-05^	99.9%
	Linear	0.71	10	-31.41					

### Changes in sex differences in swimming performance across the years

**Figure 3:**
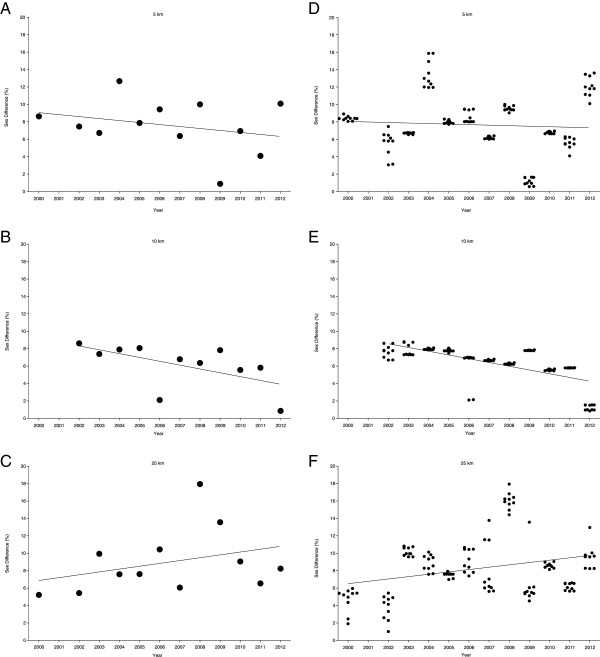
Changes in sex differences across years for the annual fastest women and men in 5 km (Panel A), 10 km (Panel B) and 25 km (Panel C) and for the annual ten fastest women and men in 5 km (Panel D), 10 km (Panel E) and 25 km (Panel F).

For the annual fastest, the sex difference in swimming speed remained unchanged in 5 km (7.6 ± 3.0%) (Figure 3A), in 10 km (6.1 ± 2.5%) (Figure 3B) and in 25 km (9.0 ± 3.7%) (Figure 3C) also when controlled for multiple finishes (Table 5). For the annual ten fastest, the sex difference remained stable in 5 km at 7.65 ± 0.59% (Figure 3D), decreased significantly and linearly (Table 6) in 10 km from 7.7 ± 0.7% (2002) to 1.2 ± 0.3% (2012) (Figure 3E) and increased significantly and linearly (Table 6) from 4.7 ± 1.4% to 9.6 ± 1.5% in 25 km (Figure 3F) also when corrected for multiple finishes (Table 5). For the annual ten fastest, the sex difference was stable at 7.6 ± 0.6% in 5 km, decreased significantly and linearly (Table 6) from 7.7 ± 0.7% (2002) to 1.2 ± 0.3% (2012) in 10 km and increased significantly and linearly from 4.7 ± 1.4% to 9.6 ± 1.5% in 25 km.

**Table 5:** Multi-level regression analyses for sex difference in swimming speed of the annual fastest and the annual ten fastest swimmers (Model 1) with correction for multiple finishes (Model 2) and age of the athletes with multiple finishes (Model 3)

**Model**	***ß***	**SE ( *ß *)**	**Stand. *ß***	**T**	***p***
		**Annual fastest swimmers**			
**5 km**					
**1**	-0.227	0.247	-0.280	-0.921	0.379
**2**	-0.227	0.247	-0.280	-0.921	0.379
**3**	-0.419	0.259	-0.515	-1.615	0.141
**10 km**					
**1**	-0.439	0.204	-0.583	-2.153	0.060
**2**	-0.439	0.204	-0.583	-2.153	0.060
**3**	-0.468	0.214	-0.621	-2.183	0.061
**25 km**					
**1**	0.326	0.294	0.331	1.111	0.293
**2**	0.326	0.294	0.331	1.111	0.293
**3**	0.330	0.311	0.336	1.062	0.316
**Annual ten fastest swimmers**					
**5 km**					
**1**	-0.060	0.081	-0.069	-0.750	0.455
**2**	-0.060	0.081	-0.069	-0.750	0.455
**3**	-0.058	0.081	-0.067	-0.718	0.474
**10 km**					
**1**	-0.430	0.043	-0.690	-9.895	< 0.001
**2**	-0.430	0.043	-0.690	-9.895	< 0.001
**3**	-0.432	0.045	-0.693	-9.702	< 0.001
**25 km**					
**1**	0.269	0.083	0.289	3.264	0.001
**2**	0.269	0.083	0.289	3.264	0.001
**3**	0.247	0.082	0.265	3.001	0.003

**Table 6:** Comparison of linear and non-linear regression analysis of changes in sex difference across years to determine which model is the best

**Sex difference**	**Kind of regression**	**Sum of squares**	**DOF**	**AICc**	**Best regression AIC-Test**	**Best regression F-Test**	**Delta**	**Probability**	**Likelihood**
Annual fastest 5 km	Polynomial	58.22	0	40.95	Linear	Undetermined	13.80	0.0010	99.8%
	Linear	94.36	10	27.14					
Annual fastest 10 km	Polynomial	21.10	0	27.16	Linear	Undetermined	10.19	0.0060	99.3%
	Linear	41.18	9	16.96					
Annual fastest 25 km	Polynomial	80.84	0	44.89	Linear	Undetermined	13.58	0.0011	99.8%
	Linear	133.50	10	31.31					
Annual 10 fastest 5 km	Polynomial	81.01	0	44.91	Linear	Undetermined	14.82	0.00060	99.9%
	Linear	120.58	10	30.08					
Annual 10 fastest 10 km	Polynomial	8.39	0	17.02	Linear	Undetermined	2.93	0.18	81.2%
	Linear	31.70	9	14.08					
Annual 10 fastest 25 km	Polynomial	119.43	0	49.57	Linear	Linear	20.29	3.9 e^-05^	99.9%
	Linear	112.67	10	29.27					

### Power density in swimming performance in finishers

**Figure 4:**
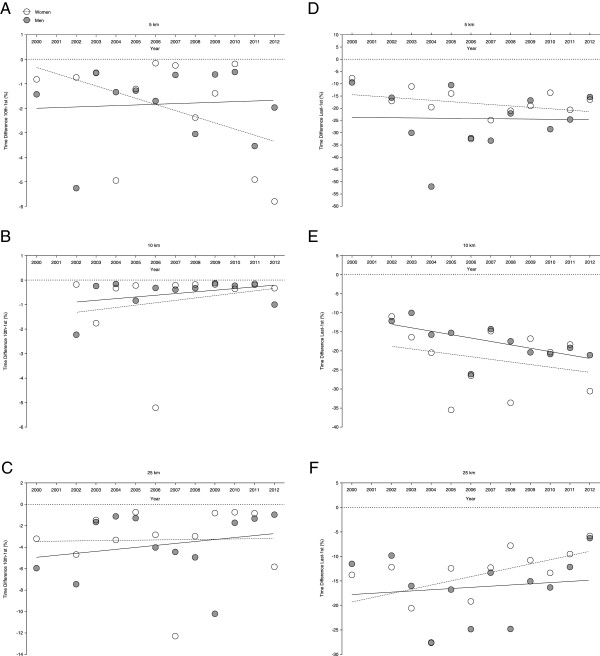
Power densities from the 10th to the 1st finisher in women and men in 5 km (Panel A), 10 km (Panel B) and 25 km (Panel C) and from the last to the 1st finisher in women and men in 5 km (Panel D), 10 km (Panel E) and 25 km (Panel F).

The power density in swimming speed of the 1st to the 10th finisher showed no changes in 5 km (Figure 4A), 10 km (Figure 4B), and 25 km (Figure 4C), also when corrected for multiple finishes and age of the athletes with multiple finishes (Table 7). Also for the 1st to the last finisher, no changes in power density were found in 5 km (Figure 4D), 10 km (Figure 4E), and 25 km (Figure 4F), also when corrected for multiple finishes and age of the athletes with multiple finishes (Table 7). The power density from the 1st to the 10th finisher was similar in 5 km (-1.95 ± 2.10% for women and -1.83 ± 1.44% for men), 10 km (-0.83 ± 1.52% for women and -0.55 ± 0.62% for men) and 25 km (-3.31 ± 3.27% for women and -3.76 ± 2.97% for men). For the 1st to the last finisher, the power density was lower in women (-18.13 ± 6.54%) compared to men (-24.21 ± 12.0%) in 5 km, higher in women (-22.21 ± 8.12%) compared to men (-17.51 ± 4.60%) in 10 km and lower in women (-13.77 ± 6.01%) compared to men (-16.22 ± 6.51%) in 25 km.

**Table 7:** Multi-level regression analyses for power density from the first to the tenth finisher and from the first to the last finisher (Model 1) with correction for multiple finishes (Model 2) and age of the athletes with multiple finishes (Model 3)

**Model**	***ß***	**SE ( *ß *)**	**Stand. *ß***	**T**	***p***
**Power density from the first to tenth finisher**					
**5 km women**					
**1**	-0.251	0.156	-0.454	-1.610	0.139
**2**	-0.251	0.156	-0.454	-1.610	0.139
**3**	-0.231	0.208	-0.418	-1.113	0.295
**5 km men**					
**1**	0.027	0.122	0.070	0.222	0.829
**2**	0.027	0.122	0.070	0.222	0.829
**3**	0.045	0.135	0.117	0.336	0.745
**10 km women**					
**1**	0.097	0.150	0.212	0.650	0.532
**2**	0.097	0.150	0.212	0.650	0.532
**3**	0.160	0.164	0.348	0.974	0.359
**10 km men**					
**1**	0.069	0.058	0.365	1.176	0.270
**2**	0.069	0.058	0.365	1.176	0.270
**3**	0.027	0.087	0.143	0.308	0.766
**25 km women**					
**1**	0.023	0.276	0.026	0.082	0.936
**2**	0.023	0.276	0.026	0.082	0.936
**3**	0.057	0.290	0.066	0.197	0.848
**25 km men**					
**1**	0.184	0.244	0.232	0.755	0.468
**2**	0.184	0.244	0.232	0.755	0.468
**3**	0.199	0.287	0.251	.0695	0.505
**Power density from the first to last finisher**					
**5 km women**					
**1**	-0.576	0.520	-0.330	-1.107	0.294
**2**	-0.576	0.520	-0.330	-1.107	0.294
**3**	-0.442	0.536	-0.254	-0.825	0.431
**5 km men**					
**1**	-0.079	1.010	-0.025	-0.078	0.940
**2**	-0.079	1.010	-0.025	-0.078	0.940
**3**	-0.005	1.118	-0.002	-0.005	0.996
**10 km women**					
**1**	-0.683	0.785	-0.279	-0.871	0.407
**2**	-0.683	0.785	-0.279	-0.871	0.407
**3**	-0.915	10.056	-0.373	-0.867	0.411
**10 km men**					
**1**	-0.893	0.354	-0.644	-2.523	0.033
**2**	-0.893	0.354	-0.644	-2.523	0.033
**3**	-10.069	0.881	-0.770	-1.214	0.260
**25 km women**					
**1**	0.860	0.428	0.537	20.013	0.072
**2**	0.860	0.428	0.537	20.013	0.072
**3**	0.861	0.453	0.537	1.901	0.090
**25 km men**					
**1**	0.243	0.544	0.140	0.447	0.664
**2**	0.243	0.544	0.140	0.447	0.664
**3**	0.159	0.532	0.092	0.298	0.772

### Change in the age of peak swimming speed

**Figure 5:**
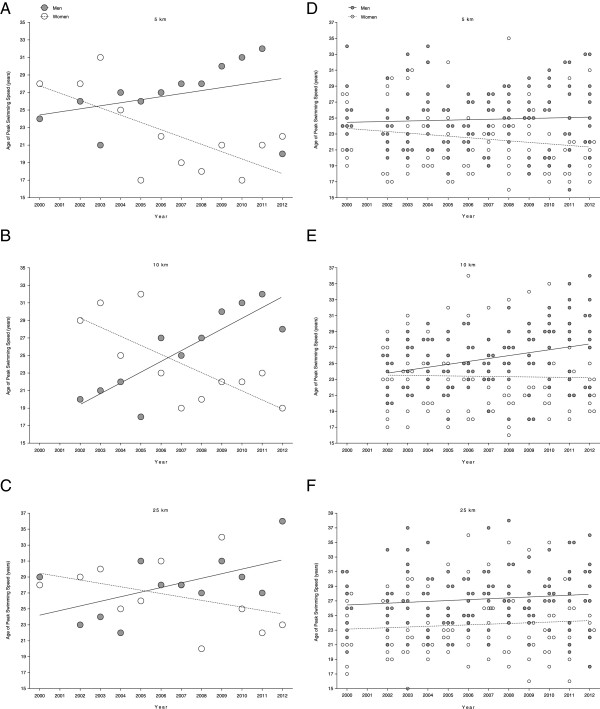
Changes in age of the annual fastest women and men in 5 km (Panel A), 10 km (Panel B) and 25 km (Panel C) and of the annual ten fastest women and men in 5 km (Panel D), 10 km (Panel E) and 25 km (Panel F).

For the annual fastest in 5 km, the age of the peak swimming speed decreased in women (Figure 5A) from 28 years to 22 years whereas it remained unchanged in men at 26.7 ± 3.6 years (Table 8). In 10 km (Figure 5B), the age of the fastest women decreased from 29 years (2002) to 19 years (Table 8). For the fastest men, however, it increased from 20 years (2002) to 28 years. In 25 km, the age of the fastest women and men (Figure 5C) remained unchanged at 26.7 ± 4.0 and 27.9 ± 3.8 years, respectively (Table 8). For the annual ten fastest, the age of peak swimming speed remained unchanged for women and men in 5 km (Figure 5D) at 22.4 ± 1.2 and 24.8 ± 0.9 years (Table 8). In 10 km, the age of the fastest women remained unchanged at 23.4 ± 0.9 years (Figure 5E) but increased in men from 24.2 ± 2.6 to 28.4 ± 4.8 years (Table 8). In 25 km (Figure 5F), the age of the fastest women and men remained unchanged at 23.7 ± 0.9 and 27.2 ± 1.1 years, respectively (Table 8).

**Table 8:** Multi-level regression analyses for change of the age of the annual fastest and the annual ten fastest swimmers (Model 1) with correction for multiple finishes (Model 2)

**Model**	***ß***	**SE ( *ß *)**	**Stand. *ß***	**T**	***p***
		**Annual fastest swimmers**			
**5 km women**					
**1**	-0.833	0.289	-0.674	-2.884	0.016
**2**	-0.833	0.289	-0.674	-2.884	0.016
**5 km men**					
**1**	0.346	0.288	0.356	1.205	0.256
**2**	0.346	0.288	0.356	1.205	0.256
**10 km women**					
**1**	-1.036	0.313	-0.741	-3.315	0.009
**2**	-1.036	0.313	-0.741	-3.315	0.009
**10 km men**					
**1**	1.227	0.240	0.862	5.113	0.001
**2**	1.227	0.240	0.862	5.113	0.001
**25 km women**					
**1**	-0.424	0.312	-0.396	-1.362	0.203
**2**	-0.424	0.312	-0.396	-1.362	0.203
**25 km men**					
**1**	0.577	0.268	0.563	2.153	0.057
**2**	0.577	0.268	0.563	2.153	0.057
**Annual ten fastest swimmers**					
**5 km women**					
**1**	-0.194	0.102	-0.172	-1.901	0.060
**2**	-0.194	0.102	-0.172	-1.901	0.060
**5 km men**					
**1**	0.054	0.098	0.051	0.552	0.582
**2**	0.054	0.098	0.051	0.552	0.582
**10 km women**					
**1**	-0.032	0.129	-0.024	-0.247	0.805
**2**	-0.032	0.129	-0.024	-0.247	0.805
**10 km men**					
**1**	0.365	0.109	0.306	3.344	0.001
**2**	0.365	0.109	0.306	3.344	0.001
**25 km women**					
**1**	0.097	0.106	0.084	0.913	0.363
**2**	0.097	0.106	0.084	0.913	0.363
**25 km men**					
**1**	0.122	0.103	0.109	1.188	0.237
**2**	0.122	0.103	0.109	1.188	0.237

## Discussion

This study investigated the changes in sex differences in ultra-swimming performance across years with increasing race distance from 5 km to 25 km and it was hypothesized that men would be faster than women from 5 km to 25 km and the sex difference in performance would be lowest in the longest race distances. As hypothesized, men were always faster than women for all distances. Women improved in 10 km but impaired in 25 km leading to a linear decrease in sex difference in 10 km and a linear increase in sex difference in 25 km. The linear changes in sex differences suggest that women will improve in the near future in 10 km, but not in 25 km.

### Participation in 5 km, 10 km and 25 km FINA World Cup races

The numbers of participants increased for men in 5 km and for both sexes in 10 km whereas the numbers of participants were constant in all other distances. Most swimmers competed and finished in 5 km, followed by 10 km and 25 km. As FINA World Cup races are organized on a professional base, *i.e.* allowing only a reserved number of participants [15] and a great increase in the numbers of participants were not to be expected. From each sex, the five fastest finishers of last year’s World Cup races and the five fastest Olympians can enter each year a series of races. Nevertheless, an increasing number of races each year provides for more possible participants. Furthermore, the number of open-water ultra-distance swimming events is small compared to indoor pool swimming [15].

### Changes in swimming speeds across years

An interesting finding was that swimming speeds remained stable across years for the annual fastest swimmers in 5 km, 10 km and 25 km. As the sex difference in swimming speed was constant in the annual fastest finishers, neither a trend of increase nor decrease in swimming speed was found nor it seems as performance in the best long-distance swimmers competing from 5 km to 25 km has plateaued. A possible explanation may by the short period of time investigated of 13 years, as other authors found an increase in swimming speed in long-distance swimming over longer periods [5-7,28]. For example, Nevill et al. reported an increase for swimming speeds in pool swimmers competing across all distances during the 60’s and 70’s when investigating data from 1956–2006, while swimming speeds plateaued in the last thirty years [28]. Therefore, swimming speed in long-distance may level off as short- and middle-distance swimming did 35 years ago.

### Sex differences in swimming speeds of the annual fastest swimmers

The best models to describe the changes in sex differences in swimming speed were linear in both the annual fastest and the annual ten fastest competitors. It has been stated that linear models cannot keep up with the gender gap in sport and non-linear models would be better [27]. Our findings, however, cannot support this theory. For the annual fastest swimmers, the sex differences remained unchanged in 5 km, in 10 km and in 25 km.

Performance in all endurance sports depends on the athlete’s ability to produce a high energy output constantly on an economical level. Both physiological and anthropometric differences between sexes seem to influence the sex difference in performance. Maximum oxygen uptake (VO_2_max) was reported as the most significant predictor variable for endurance performance [29]. While elite male athletes reach a VO_2_max of up to 85 ml · min^-1^ · kg^-1^[30], women’s VO_2_max is lower with a maximum of 70 ml · min^-1^ · kg^-1^[31]. VO_2_max is mainly dependent from the heart’s performance and the lung capacity [32]. The maximal cardiac output in elite male athletes is higher than in elite female athletes [33]. The same was reported for lung capacity [34]. VO_2_max directly depends from both maximal cardiac output and lung capacity and is therefore larger in men than in women [32]. Therefore, men have generally more physiological potential to perform at a higher level than women.

The sex difference in performance was often discussed in other sports disciplines such as running [35,36], cycling [37], swimming [5-7], or the combination of the three in triathlon [4,38]. The probably most controversial discussed publication dates back to 1992, when Whipp and Ward [35] used linear statistical models to prove their point of women outrunning men in marathon running. A series of publications followed, discussing the subject intensely [27,36,39,40]. So far, in neither of the mentioned sports, women were ever able to outperform men with very rare single exceptions such as the initially mentioned Diana Nyad swimming from Cuba to Florida [23]. In triathlons from the classical Olympic distance (*i.e.* 1.5 km swimming, 40 km cycling and 10 km running) [41] to Deca Iron ultra-triathlons (*i.e.* 38 km swimming, 1,800 km cycling, 422 km running) [22], the authors found sex differences of performance that seemed to increase with increasing duration or distance of the triathlon event.

Swimming speed results in single open-water long-distance performances of Diana Nyad and Christoph Wandratsch are hard to compare as water temperatures and weather cannot be influenced but have an influence on performance [7,42]. Effects of water temperature and different means to avoid undercooling or overheating have been discussed differently [43]. Both undercooling [43] and overheating [44] threaten performance after prolonged exposure. However, specific anthropometric characteristics such as body fat seemed to influence performance in long-distance swimming. A high body fat percentage was favourable of withstanding the cold of the water [45,46]. To avoid large differences between different races, the highest as well as the lowest temperature in long-distance swimming are defined by FINA. Above 31°C and below 16°C there are no races at all, between 16°C and 26°C swimmers may use wetsuits [15].

### Differences in performance and sex differences in performance for the annual ten fastest swimmers

In the annual ten fastest, women became faster in 10 km but slower in 25 km whereas men showed no changes in performance. These changes in female performances led to a linear decrease in sex difference in 10 km and a linear increase in sex difference in 25 km. In the annual fastest, however, no changes occurred in both swimming speeds and sex differences in swimming speeds. Therefore, women could not generally reduce the gender gap in ultra-distance swimming performance, especially in the longer race distances.

In contrast to the annual fastest swimmers, the annual ten fastest women became faster in 10 km between 2000 and 2012. Maybe the 10 km race is an ideal race for women. A recent study investigating the performance in 10 km open-water swimming including World Cup races, European Championships, World Championships and Olympic Games from 2008 to 2012 showed an unchanged swimming speed for women but performance impaired in men [9]. A possible explanation may be drafting as the 10 km could be the optimal distance (*i.e.* swimming speed and duration of the race) to draft a whole race. In 5 km, the sex difference in muscle strength may allow men to outpace women whereas 25 km may be too long to simply rely on drafting for women. However, other unknown factors may be present in 10 km. As our results are in line with findings for recreational athletes [5-7] it seems rather unlikely that women will outperform men in the future in longer swim distances. This assumption will be supported by the unchanged power density in swimming speed for both women and men.

The sex difference in performance between female and male endurance and ultra-endurance athletes might be partially explained by differences in anthropometric characteristics between women and men such as differences in skeletal muscle mass and body fat. Knechtle et al. [22] argued that the increase in sex difference with increasing race distance in ultra-races such as an ultra-triathlon was most probably due to the lower skeletal muscle mass in women. It has been shown that male ultra-endurance athletes had a higher skeletal muscle mass than female ultra-endurance athletes [47-51]. For example, male Ironman triathletes with 41 kg skeletal muscle mass had a 46% higher skeletal muscle mass compared to female Ironman triathletes with 28 kg skeletal muscle mass [48]. Considering ultra-runners, male ultra-runners with 38 kg skeletal muscle mass [49] had a 38% higher muscle mass compared to female ultra-runners with 27.4 kg [50]. For ultra-swimmers [51], the sex difference in skeletal muscle mass was considerably higher compared to runners [49,50]. Male open-water ultra-swimmers with 42 kg of skeletal muscle mass had 45% more skeletal muscle mass compared to female open-water ultra-swimmers with 29 kg of skeletal muscle mass [51]. These differences in skeletal muscle mass between female and male ultra-endurance athletes might explain the increase in sex difference with increasing race length in open-water ultra-distance swimmers.

### The age of peak performance in elite long-distance swimmers

The age of peak swimming speed in these elite open-water ultra-swimmers was at ~22-28 years depending upon the race distance. These swimmers were therefore considerably younger compared to recreational swimmers investigated by Eichenberger et al. [6,7] where the age of peak swimming speed was at ~30-39 years in recreational 12-hour pool swimmers [6] and recreational 26.4 km open-water swimmers [7]. In pool swimming, elite athletes were younger than open-water ultra-swimmers as reported by Berthelot et al. [52] with ~21 years for elite pool-swimmers competing in 50 m to 1,500 m freestyle. However, Berthelot et al. [52] reported differences in the age of peak swimming speed regarding the length of a race. The peak performance in 1,500 m freestyle was achieved at a younger age of ~18 years compared to the 50 m freestyle at ~23 years, respectively [52]. In elite freestyle swimmers competing at national level, the age of peak swimming speed was at ~19-25 years when all distances were considered [21]. In 50 m freestyle, women were fastest at the age of ~20 years and men at the age of ~23 years. Considering the longest pool distance, women were fastest in 1,500 m freestyle at the age of ~18 years and men at the age of ~20 years [21].

Considering the results of the present study it seems likely that the age of peak swimming speed decreased from ~20-23 years in of 50 m to ~18-20 years in 1,500 m to increase again to ~23-27 years in 25 km events. Recent investigations [53,54] addressed a potential association of the age of peak performance with the length of an event. In ultra-marathon running, Rüst et al. [54] mentioned the possibility that older runners rather compete in ultra-marathons due to a deficit in physiological factors such as VO_2_max compared to young athletes in their best age. It could be argued that the world’s elite swimmers at the age of ~20 years rather compete in short- and middle distance than in long-distance events. They may change to the ultra-distances after their career in the short distances.

Physiological and anthropometric differences might explain the differences in the age of peak performance between short- to middle-distance and long-distance swimming. Peak swimming speed in sprint swimming was highly associated to strength, power [55] and anaerobic capacity [56]. In longer race distances such as the 1,500 m freestyle, peak swimming speed was rather associated with VO_2_max [56], anaerobic threshold [57] and anthropometric characteristics such as body fat [58-60].

### Strength, limitations and implications for future research

The study is the first to analyse the sex difference of performance in professional open-water long-distance swimmers competing in the FINA World Cup races in 5 km, 10 km and 25 km. The strength of the study is that the statistical analysis excluded the influence of multiple finishes of the same athlete and bypassed a therefore a possible bias of results. Furthermore, both linear and non-linear analyses were performed to find the best model for each point of interest. The study is limited since variables such as physiological parameters [61], anthropometric characteristics [62], training data [62], previous experience [63], nutrition [64,65] and motivational [66] factors were not considered but may have influenced the results. Further studies need to investigate why swimming speeds in elite long-distance swimming plateaued from 2000–2012 and why the sex difference of performance in long-distance swimming was smaller than expected. Moreover, a systemic analysis of the sex difference of swimming speed across distances from 50 m to 25 km and more would give further insights in the sex difference for different race distances. Future studies might investigate from which country the fastest swimmers in 5 km, 10 km and 25 km originate.

### Practical applications

For athletes and coaches, women showed no changes in swimming speed in the 5 km FINA races, increased swimming speed in the 10 km FINA races but decreased swimming speed in the 25 km FINA races. The fastest swimming speeds were attained at the age of ~22-28 years considering all distances from 5 km to 25 km. Elite women intending to improve in 25 km and to lower the gender gap in 25 km would most probably need to increase skeletal muscle mass and muscular strength to follow the fastest men.

## Conclusions

To summarize, elite female swimmers showed a linear improvement in swimming speed in 10 km but a linear impairment in swimming speed in 25 km leading to a linear decrease in sex difference in 10 km and a linear increase in sex difference in 25 km. The linear changes in sex difference in swimming speed suggest that women will improve in the near future in 10 km, but not in 25 km. It is very likely that the gender gap to men will be further reduced in 10 km but it is very unlikely that the gender gap will be reduced in 25 km.

## Competing interests

The authors report no conflicts of interest.

## Authors’ contributions

MZ drafted the manuscript, CR performed the statistical analyses, TR and RL participated in the design of the study and helped drafting the manuscript, BK collected the data, helped in interpretation of the results and drafting the manuscript. All authors read and approved the final manuscript.

## Pre-publication history

The pre-publication history for this paper can be accessed here:

http://www.biomedcentral.com/2052-1847/6/7/prepub
